# Prescribing and Monitoring of Antipsychotics for Behavioural and Psychological Symptoms of Dementia in a Community Older Adult Mental Health Service: A Retrospective Audit

**DOI:** 10.1192/bjo.2026.11707

**Published:** 2026-06-30

**Authors:** Silvester Hammed, Rachel Wright, Laura Davis, Rob Tomlinson, Anjana Sathian

**Affiliations:** Nottinghamshire Healthcare NHS Foundation Trust, Nottingham, United Kingdom

## Abstract

**Aims:**

Antipsychotics may be prescribed for severe behavioural and psychological symptoms of dementia (BPSD) but carry significant risks. National and local guidance recommends structured assessment, documentation, regular review, and timely deprescribing.

This audit assessed compliance with Nottingham Area Prescribing Committee guidance EG019 and National Institute for Health and Care Excellence guidance NG97 within the Newark and Sherwood Older Adult Community Mental Health Team.

We hypothesised that initial clinical assessment would show high compliance, while structured monitoring documentation would be inconsistent.

**Methods:**

A retrospective audit was conducted using the RiO electronic clinical record system. A Clinical Record Interactive Search identified patients with a diagnosis of dementia initiated on antipsychotic medication by the Newark and Sherwood Older Adult Community Mental Health Team between 1 October 2023 and 1 October 2024. Diagnoses included International Classification of Diseases, Tenth Revision (ICD-10) codes F00–F04 and G31.8.

The audit tool was a locally developed audit proforma derived from the standards from the two guidances (EG019 and NG97), and it assessed documentation of symptom assessment, risk evaluation, discussion of risks and benefits, use of the Antipsychotics in Dementia Assessment and Monitoring Form, review intervals, and consideration of deprescribing. Compliance was reported descriptively as percentages.

**Results:**

Of 114 records screened, six met inclusion criteria.

- Target symptoms were identified and quantified in 100% of cases, and contributing factors for distress were explored in all patients.
- Delirium was considered in 83%, and modifiable factors were addressed in 83%.
- Lewy body or Parkinson’s disease dementia was considered in all cases.
- Risk of harm to self or others was documented in 67%
- Discussion of risks and benefits with patients or carers was documented in 83%.
- Medication review occurred in 100%, with dose reduction or discontinuation in 17%.
- Review at or before six weeks occurred in 80% of eligible cases, with a documented rationale for continuation in 60%.
- Regular six-weekly review and consideration of deprescribing were each evident in 60%.
- Notably, the completion of the Trust Antipsychotics in Dementia Assessment and Monitoring Form occurred in 0%.

**Conclusion:**

This audit demonstrates good compliance with recommended clinical assessment and cautious prescribing for BPSD but identifies a critical gap in structured monitoring, with no use of the Trust-mandated monitoring form. Documentation of ongoing review and rationale for continuation was also inconsistent. These findings support targeted education, improved induction processes, and system-level changes to embed structured monitoring. A re-audit, including baseline physical health monitoring, is planned.

